# Diagnosis of Cardiac Abnormalities in Muscular Dystrophies

**DOI:** 10.3390/medicina57050488

**Published:** 2021-05-12

**Authors:** Elisabeta Bădilă, Iulia Ioana Lungu, Alexandru Mihai Grumezescu, Alexandru Scafa Udriște

**Affiliations:** 1 “Carol Davila” University of Medicine and Pharmacy, 050474 Bucharest, Romania; elisabeta.badila@gmail.com (E.B.); alexscafa@yahoo.com (A.S.U.); 2 Internal Medicine Department, Clinical Emergency Hospital Bucharest, 014461 Bucharest, Romania; 3 Faculty of Applied Chemistry and Materials Science, University Politehnica of Bucharest, 011061 Bucharest, Romania; grumezescu@yahoo.com; 4 Research Institute of the University of Bucharest—ICUB, University of Bucharest, 050657 Bucharest, Romania; 5 Cardiology Department, Clinical Emergency Hospital Bucharest, 014461 Bucharest, Romania

**Keywords:** neuromuscular disorders, cardiac abnormalities, muscular dystrophies, cardiac imaging

## Abstract

Muscular disorders are mainly characterized by progressive skeletal muscle weakness. There are several aspects that can be monitored, which are used to differentiate between the types of muscular disorders, ranging from the targeted muscle up to the mutated gene. An aspect that holds critical importance when managing muscular dystrophies is that most of them exhibit cardiac abnormalities. Therefore, cardiac imaging is an essential part of muscular disorder monitoring and management. In the first section of the review, several cardiac abnormalities are introduced; afterward, different muscular dystrophies’ pathogenesis is presented. Not all muscular dystrophies necessarily present cardiac involvement; however, the ones that do are linked with the cardiac abnormalities described in the first section. Moreover, studies from the last 3 years on muscular disorders are presented alongside imaging techniques used to determine cardiac abnormalities.

## 1. Introduction

Muscular dystrophies are hereditary disorders that are characterized by gradual and continuous skeletal muscle weakness. Several aspects need to be taken into account in order to establish a clinical diagnosis; these features include but are not limited to the severity of the muscular wasting, as well as its distribution, and the accompanying symptomology, biochemical, hematological, physical, and neurological investigations, electromyography, and muscle biopsy. Moreover, if the gene defect is established, diagnosis can also be confirmed by gene testing [1,2].

Cardiac diseases have been observed in neuromuscular disorders, distinctly muscular dystrophies [3,4,5]. However, cardiac diseases’ involvement does not relate to the severity or advancement of the disorder, and it can be either the initial symptom or even the dominating one. Sudden death and progressive heart failure are the main causes that determine cardiac death. Due to the advancement in medical management over the years, patients’ survival rate suffering from muscular dystrophy is significantly increased. However, one main aspect that contributes to mortality is cardiac involvement [2].

## 2. Cardiac Abnormalities

### 2.1. Cardiomyopathies

Cardiomyopathies are described as myocardial diseases in which the structure and the behavior of the heart are abnormal. Since cardiomyopathies are mainly characterized by the left ventricle’s dimensions and function, imaging techniques, such as echocardiography and cardiac magnetic resonance, can be used for diagnostic purposes [6].

#### 2.1.1. Dilated Cardiomyopathy (DCM)

Dilated cardiomyopathy is characterized by left ventricular dilatation, as well as the apparition of contractile dysfunction. The right ventricle may also be dilated and dysfunctional. DCM has been classified as being one of the main reasons for heart failure worldwide. It has been statistically observed that children with DCM under the age of one year have the highest incidence for this disorder [4]. Although the cause for DCM is still uncertain, it was observed that it can be genetic, due to ischemic damage or acquired (for example, alcohol, and methamphetamine abuse) [7]. Sudden death occurs in over 10% of the patients with dilated cardiomyopathy; however, the mortality rate is much higher in children compared to adults [8].

#### 2.1.2. Hypertrophic Cardiomyopathy (HCM)

Hypertrophic cardiomyopathy can be described as a genetic disease of the cardiomyocytes, which is characterized by asymmetrical cardiac hypertrophy. HCM does not have a clear ethnic or gender-related distribution. Unfortunately, patients suffering from HCM usually present minimal symptoms or are asymptomatic. However, HCM can lead to sudden cardiac death (most commonly in competitive athletes) and heart failure, the majority of patients suffering from HCM lead a normal life [9].

#### 2.1.3. Restrictive Cardiomyopathy (RCM)

Restrictive cardiomyopathy is characterized by a diastolic dysfunction of the left or right ventricle, which are non-dilated. Ventricular chambers size and systolic function are usually normal or near-normal until later stages of the disease. Due to the fact that it can affect either one or both ventricles, the patients with this disorder may have symptoms of heart failure, but also arrhythmias and conduction disturbances. The pathogenesis of RCM can be hereditary, acquired, or mixed. Due to the difficulties in diagnosing RCM, it is challenging to estimate its predominance and occurrence; however, amongst all cardiomyopathies, it has been determined that it has the lowest incidence [10].

#### 2.1.4. Left Ventricular Non-Compaction Cardiomyopathy (LVNC)

Left ventricular non-compaction cardiomyopathy is a rare genetic disorder distinguished by uncommon and excessive trabeculations found in the left ventricle. It is considered that during cardiac development, the heart fails to form a complete myocardium. Although not as common, the right ventricle may also be affected, either by itself or at the same time as the left ventricle. Another distinguishing feature is the fact that the myocardium thickens into two separate layers comprised of compacted and noncompacted myocardium. LVNC is associated with ventricular failure, either left, right, or both. The patients suffering from LVNC are usually asymptomatic, however symptoms of heart failure can be induced mainly by exercise [11].

### 2.2. Takotsubo Syndrome (TTS)

Takotsubo syndrome is very similar in symptoms to acute coronary syndrome and can be described by severe left ventricular dysfunction from which the patient can usually recover impromptu in several days to weeks. Symptoms generally consist of chest pains or shortness of breath. It has been observed that for the majority of patients, the onset of TTS is strictly correlated with negative stressors, such as suppressed terror, physical, and emotional stress. In some rare cases, stress is not the reason for the onset of TTS [12]. Interestingly, Ghadri et al. conducted research in which the results indicated that patients with positive stressors exhibit similar symptoms to those with negative ones; therefore, they concluded that both stressors may have a role in triggering TTS [13].

## 3. Classification and Pathogenesis of Muscular Dystrophies

**Figure 1:**
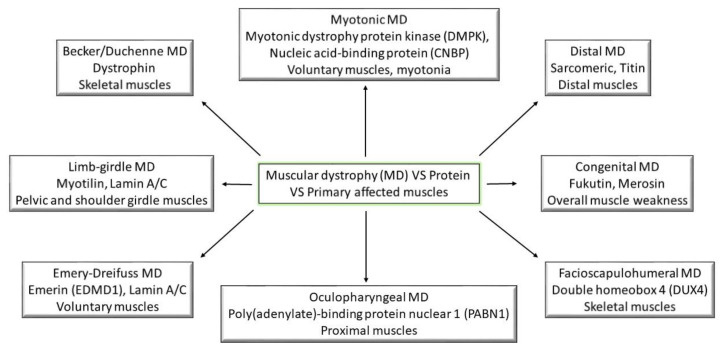
Muscular dystrophies and their primary affected proteins and muscles; (MD—muscular dystrophy).

**Figure 2:**
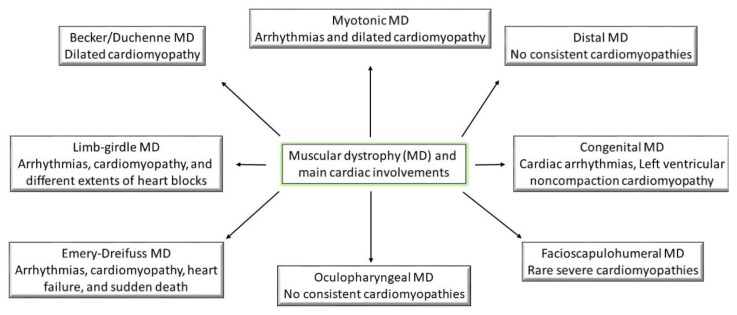
Muscular dystrophies and their main characteristic cardiac involvements.

Taking into account the symptomology recorded for all the types of muscular dystrophies, they have all reported being linked through weakness and progressive behavior (Figure 1); however, the main differences between them come from the age of symptom onset and the progression rate [1]. The following part will try to shed some light on the pathogenesis of different muscular dystrophies (Table 1). Moreover, the cardiac abnormalities presented in the previous section will be linked to muscular disorders (Figure 2).

### 3.1. Becker and Duchenne Muscular Dystrophy

Becker and Duchenne muscular dystrophies, also known as dystrophinopathies, are progressive muscle diseases that are inherited as X-linked recessive conditions. The primarily affected muscle is the skeletal one. The responsible gene for these diseases is *DMD* or the dystrophin gene, which is the largest gene in the human body, and it is in charge of encoding the dystrophin protein. This protein is mainly found in the skeletal and cardiac muscles, and its role (as part of a Dystrophin Associated Protein Complex) is to strengthen the muscle fibers as they constantly contract and relax [14]. These dystrophies almost exclusively affect males due to the fact that the *DMD* gene is located on the X chromosome; however, females can be carriers of the gene and are usually asymptomatic. The main distinction between the two dystrophinopathies is that in Becker muscular dystrophy, the dystrophin protein is partially functional, while in Duchenne muscular dystrophy, there is no dystrophin protein at all [15]. Therefore, Becker muscular dystrophy exhibits a milder phenotype; it is less common. The lack, and even the partially functional existence, of dystrophin protein leads to muscle fiber degeneration [16,17,18,19]. The most frequent cardiac abnormality in both Becker and Duchenne muscular dystrophies is dilated cardiomyopathy; however, it can have different onset ages and disease severity. Compared to the usual dilated cardiomyopathy cases, dystrophinopathies are presented with a late left ventricular dilatation, but with contractile dysfunction at onset, without any ventricular dilatation indicator [14]. Due to the motor disability caused by the skeletal muscles’ damage, there may not be any early clinical indicators of cardiac failure. However, left ventricular dilatation can be observed using cardiac imaging techniques such as echocardiograms. Yet, it has been noted that standard echography is not as useful in detecting early stages of cardiomyopathy. Studies have shown that color Tissue Doppler imaging can detect abnormal strain rates in the posterior wall of the myocardium in cases where standard echocardiography was normal for Duchenne patients. The study presented by Mori et al. also has its limitations, such as the discussed method could only be applied for patients that had a wall thickness (left ventricular posterior wall) greater than 6 mm.; inaccuracy due to lack of homogeneity of myocardial deterioration; this method depends greatly on the quality of the 2D echocardiography used [20]. Another technique that can be used in order to overcome the drawback of standard echography is Cardiac Magnetic Resonance (CMR). This technique also has the advantage of providing a non-invasive assessment of the cardiac function. Moreover, adding late gadolinium-based imaging methods can significantly improve the view over myocardial fibrosis. Brunklaus et al. reported a comparison between CMR and echocardiography assessed on 35 Duchenne muscular dystrophy patients. Their findings showed that in more than 20% of the cases, there were differences between the results, including cardiac dysfunctions that were detected by CMR, but not by echocardiography [21]. Young patients who suffer from Becker muscular dystrophy are usually asymptomatic; as they grow older, the risk increases, and most patients develop symptomatic heart failure by the age of 40 [5]. In the case of Duchenne muscular dystrophy, the main cause of mortality is cardiomyopathy; the systolic function of the left ventricle is severely impaired due to myocardial tissue injuries. There is no correlation between the types of dystrophinopathies and the evolution of cardiomyopathy. Even though female carriers are usually asymptomatic, cases of left ventricular dysfunction can be observed by inducing exercise, and some women may even develop severe heart failure [22,23].

Due to the fact that cardiomyopathy is the leading cause of morbidity in patients suffering from Duchenne muscular dystrophy, it is highly recommended that they undergo early detection. As an example, when the patient is diagnosed, initial echocardiography is carried out, and for patients below 10 years of age, an annual or biannual echography is recommended. As for senior patients, left ventricular function is evaluated annually using an echocardiogram. There are studies that present several guidelines for cardiac management and diagnosis for Duchenne muscular dystrophy patients from early to late non-ambulatory stages. These guidelines include the basic baseline for diagnosis such as cardiac medical/family history, electrocardiograms, echocardiograms, and CMR, which should also be taken as annual evaluations [24]. Based on cardiologist recommendations, the frequency of the assessments can differ from patient to patient. For ambulatory and early non-ambulatory stage patients, annual evaluations should be considered as well as pharmacological therapy based on age and symptoms. In the late non-ambulatory stage, there is a high demand for close cardiac monitorization [24]. Moreover, respiratory care should also be improved because it is strongly linked to cardiac functions. There are also cases where a left ventricular assistance device needs to be taken into account. Female carriers are also at risk for cardiomyopathy, and therefore are advised to undergo a cardiac evaluation early on with surveillance every 3 to 5 years [24]. As a link between cardiac involvement and Duchenne muscular dystrophy, it has been observed that X-linked dilated cardiomyopathy (a disease where the cardiac muscle has no dystrophin expression) is usually accompanied by normal levels of dystrophin expression in skeletal muscles. Yamamoto et al. realized a study on 181 patients in order to determine a correlation between cardiac involvement and dystrophin isoform shortage. Based on the different mutations of *DMD*, the patients were organized into several groups. The study used echocardiograms in order to determine the left ventricular ejection fraction and left ventricular end-diastolic dimension. The results indicated that the patients with a mutation in the dystrophin isoform Dp116 are prone to cardiac dysfunction, thus leading to the conclusion that suppressing this dystrophin isoform may hinder cardiac dysfunction in Duchenne muscular dystrophies patients [25].

When discussing the cardiac involvement in Becker muscular dystrophy, it has been established that it is a common feature; however, it can be symptomatic or asymptomatic, also known as subclinical. Most patients develop asymptomatic cardiac involvement, which can be identified only through further investigations. The most common asymptomatic cardiac abnormalities are dilated cardiomyopathy accompanied by heart failure. Patients that suffer from symptomatic cardiac involvement have symptoms that vary from barely any cardiac abnormalities to severe arrhythmias, hypertrophic or dilated cardiomyopathy, heart failure, or sudden cardiac death. The are several symptoms correlated to age for patients that have Becker muscular dystrophy: For the first 10 years of life, the patients are usually symptom-free, apart from elevated creatine kinase; during the next 5 years, they start to experience gait abnormality; muscle weakness steadily progresses for the following 5 years. However, only after 30 years of age, when the patients start having difficulties during manual work, do cardiac abnormalities occur [26]. There are several clinical investigations that can be used to detect symptomatic and asymptomatic cardiac abnormalities. Blood chemical investigations are used to determine the degree of myocardial cell damage, electrocardiograms detect rhythm abnormalities or myocardial damage; echocardiograms and cardiac magnetic resonance imaging can detect dilated and hypertrophic cardiomyopathies, ventricular dysfunctions, and fibrosis. Myocardial fibrosis, arrhythmias, systolic, and diastolic dysfunctions can be detected by backscatter analysis and technetium ventriculography. Moreover, magnetic resonance spectroscopy can assess the reduced expression of dystrophin, and endomyocardial biopsy is used to detect fibrosis, left ventricular hypertrabeculation, and atrophic cardiomyocytes [26].

### 3.2. Congenital Muscular Dystrophy

Congenital muscular dystrophies are inherited diseases where muscle weakness can be observed either immediately after the child is delivered or during the first six months of life. In this type of disorder, muscle weakness can either progress slowly or it can be non-progressive. The mortality rates differ from type to type; there are cases where there is minimal disability shown and the child can grow into adulthood or cases where the child can die during its early childhood. Syndromic congenital muscular dystrophy is characterized by a structural brain defect, including or not including mental retardation [27]. However, more than half of the cases do not exhibit mental retardation and are classified as classic congenital muscular dystrophies. Syndromic congenital muscular dystrophies include Fukuyama congenital muscular dystrophy, Muscle-eye-brain-disease, Walker–Warburg syndrome, and congenital muscular dystrophy type 1D (MDC1D). These are correlated with mutations of genes, such as *LAMA2, COL6A, FKTN, LARGE, POMT1,* and *CHKB*. For example, Fukuyama congenital muscular dystrophy is caused by a defective gene that encodes the Fukutin protein. Patients were suffering from this type of dystrophy present muscle weakness and mental retardation. Merosin-deficient congenital muscular dystrophy (MDC1A) is caused by a mutated gene, *LAMA2*. It has been reported that around 35% of congenital muscular dystrophy cases in European countries are MDC1A [1,2,28]. For patients suffering from MDC1A, cardiac involvement is unusual. However, one-third of the diagnosed patients present cardiac abnormalities during testing, and only a small part of them are symptomatic [29]. The occurrence of Fukuyama congenital muscular dystrophy is mainly in Japan. Infants who are affected by this disorder present impaired motor development and several mental retardations. Moreover, after the age of 10, the patients develop left ventricular dysfunction, which ultimately leads to heart failure [2]. When compared to the cardiac abnormalities in Duchenne muscular dystrophy, it has been concluded that Fukuyama congenital muscular dystrophy exhibits less severe cardiac dysfunction [30]. However, cardiac abnormalities, especially dilated cardiomyopathy, have been found to occur commonly in patients with Fukuyama congenital muscular dystrophy [31]. Cardiac involvement has been observed in patients carrying the *LMNA* mutated gene in the form of cardiac arrhythmias [32,33]. Even though cardiac involvement is not usually reported in Walker–Warburg syndrome cases, non-compaction cardiomyopathy has been associated with a case that also included several other congenital heart malformations [34].

Ullrich is a rare congenital muscular dystrophy caused by the mutation of the following genes: *COL6A1*, *COL6A2*, or *COL6A3*. This muscular dystrophy is distinguished by general muscle weakness, contractures of proximal joints, and the distal joints’ ability to move beyond their normal sphere of motion. The onset of the disease is usually at birth or quickly after that, only within one year of life. Another distinguishable feature is the decrease of respiratory functions after the age of six. Although there are several studies in this aspect, and routine clinical examinations, including electrocardiography and echocardiography, have been done on patients suffering from Ullrich muscular dystrophy, it may seem that this type of congenital muscular dystrophy does not develop cardiac involvement. Bethlem congenital muscular dystrophy is allelic to Ullrich, therefore is caused by the mutation of the same genes. It is characterized by gradual muscle weakness and wasting, deterioration in respiratory functions, joint contractures, and distal laxity. Similar to Ullrich muscular dystrophy, several studies have been done in order to determine de cardiac implication in Bethlem congenital muscular dystrophy. However, even though there were cases that presented cardiac abnormalities, there were not attributed to cardiac implications determined by the muscle disorder [29].

### 3.3. Distal Muscle Dystrophy

Distal muscle dystrophies are inherited muscle disorders that are associated with muscle weakness in a progressive manner and atrophy, usually in the hands, forearm, lower leg, and feet. Although distal muscle dystrophy has its representative gene defects, some can also generate other phenotypes. One of the most known distal muscle dystrophy is Welander distal myopathy (WDM), for which the gene defect is still unknown. It has been observed that the genes involved in this disorder are likely to affect sarcomeric proteins. Tibial muscular dystrophy, also known as Udd myopathy, affects the anterior tibialis muscle, usually after the age of 30. It is very common that the disease’s progression (which is slow) takes place in an asymmetrical manner. Even after several years, the patients do not usually require wheelchairs indefinitely. However, cardiomyopathies are not a consistent characteristic for distal muscular dystrophies [35,36].

### 3.4. Emery–Dreifuss Muscular Dystrophy

Emery–Dreifuss muscular dystrophy is an X-linked recessive disease that is characterized by several aspects: Early joint contractures of the Achilles tendon, elbows, and neck; progressive muscle weakness and atrophy, which initially has a humerus-fibula distribution but can later on extend; and cardiac abnormalities. The disease has been presented clinically in variable forms, from early-onset with severe symptoms to a late onset of the diseases presenting slow progression. Most commonly, the symptoms will start with joint contractures, usually in the first 20 years of life; the disorder will then be followed by muscle weakness and atrophy. The latter stage of the disease is usually accompanied by cardiac involvement, including impaired respiratory capacities in some cases. Emery–Dreifuss muscular dystrophy may result from *EMD* gene defect, which is related to the encoding of emerin protein (EDMD1). However, other forms may be presented, such as autosomal dominant Emery–Dreifuss muscular dystrophy (EDMD2), which is a rare form caused by *LMNA* gene defects, encoding lamin A/C. The extent to which the disease is widespread is unknown. Lamin A/C gene mutation does not only cause Emery-–Dreifuss disorder, but it has been found to determine various other phenotypes. However, distinguishing between the two Emery–Dreifuss phenotypes is clinically troublesome, which can lead to the suggestion that there may be a connection between the two proteins. It has been noted that the cases that involve dilated cardiomyopathy most commonly are cases of EDMD2 [2,37,38]. Emery–Dreifuss muscular dystrophy is known to be associated with progressive cardiac abnormalities that lead to heart blocks and sudden death [5]. The cardiac abnormalities are due to the gradual replacement process of healthy myocardium with fibrosis and adipose tissue initiating in the atria and often involving atrioventricular node and ventricles; severity is not influenced by muscular weakness progression. Arrhythmia, cardiomyopathy, atrioventricular block, strokes, heart failure, and sudden death have all been reported for patients suffering from Emery–Dreifuss muscular dystrophy [39,40]. Moreover, for patients suffering from Lamin A/C mutations, the cardiac features are very commonly associated with atrioventricular conduction irregularities, as well as life-threatening cardiac arrhythmias [41].

A research study highlights the importance of engineered mouse models of Emery–Dreifuss muscular dystrophy in order to identify the warning pathways accountable for cardiac abnormalities. For example, a specific mouse model that had the mutation of the gene *LMNA* p.H222P offered an insight on cardiac involvement, associating the development of dilated cardiomyopathy with cardiac conduction defects [31].

Other associated genes can be *SYNE1* and *SYNE2* encoding nesprin-1 and nesprin-2, *FHL1* encoding FHL1, *TMEM43* encoding LUMA, *SUN1* and *SUN2* encoding SUN1 and SUN2 and *TTN* encoding titin. Even though *SYNE1* mutations do not come with significant cardiac involvement, *SYNE2* generally does, ranging from arrythmias to cardiac failure. Cardiac involvement is also usually present in *FHL1* mutations, though it usually develops after skeletal muscle manifestation and in *TMEM43* mutations [42,43]. Moreover, it has been shown that the gene *TMEM43* is associated with a unique form of arrhythmogenic right ventricular cardiomyopathy (ARVC) [44].

### 3.5. Facioscapulohumeral Muscular Dystrophy

Facioscapulohumeral muscular dystrophy is a distinctive disorder with a clear pattern of skeletal muscle weakness and atrophy and an extensive range of disease severity. As the name indicates, the main muscles affected are the face, shoulder blades, and upper arm. Symptomology usually installs in adolescence; however, there are cases where the diseases is not noticeable until adulthood and cases with high severity that can be observed since infancy. The progressive muscle weakness can spread to other parts of the body, such as the lower leg, abdominal, hip, and pelvis muscles. Due to its increased prevalence, facioscapulohumeral muscular dystrophy is recorded to be in the top five most common muscle disorders, falling right under Duchenne and myotonic dystrophies [32]. This disease, however, does not have lethal pulmonary or cardiac involvements, therefore the life span of the patients is usually not affected. There have been two types recorded: Facioscapulohumeral muscular dystrophy type 1 (FSHD1) and facioscapulohumeral muscular dystrophy type 2 (FSHD2); both having the same symptomology, they can be distinguished through genetic testing. This dystrophy’s pathophysiology is related to a modification of an area of DNA close to the end of the chromosome D4Z4. It is not common for patients to present respiratory or cardiac involvement. However, cases of patients with restrictive lung disease have been reported for severe cases where the patient is usually a wheelchair user [45,46]. A recent study on 56 adult patients with facioscapulohumeral dystrophy type 1 was conducted in order to determine cardiac involvement. It was observed that around 50% of the patients have minor cardiac involvement, with the most frequent one being incomplete right bundle branch block. After a 7-year follow-up, there was still no significant cardiac involvement, including only minor abnormalities [47].

### 3.6. Limb–Girdle Muscular Dystrophy

Limb–girdle muscular dystrophy is a heterogeneous disorder distinguishable pelvic and shoulder girdle muscles weakness. There are two types of limb–girdle muscular dystrophies identified, one is autosomal dominant LGMD1 and autosomal recessive LGMD2, each having several subtypes. Up to this day, over 30 genetic types of limb–girdle muscular dystrophies have been identified. It has been noticed that the incidence of LGMD2 is much higher than LGMD1, and some subtypes of LGMD1 are found in single families due to individual mutations. Symptoms include distal and central muscle weakness and selective muscle atrophy, however facial, mouth, and throat muscles are generally not harmed. One of the differences between the two types of limb–girdle muscular dystrophy is the onset time, where in the case of LGMD1 the onset of the diseases is usually in the early ages, while LGMD2 is known to have an adult-onset [48]. Due to the different types and subtypes, the disorder has variable progressive conditions; however, severe cases involve cardiac and pulmonary abnormalities, leading to lower life expectancy. The main cardiac abnormalities involved in patients who have limb–girdle muscular dystrophy include arrhythmia, cardiomyopathy, and different extents of heart blocks. It is highly recommended for patients with conduction abnormalities to routinely undergo electrocardiography and echocardiography [5,48,49].

The extent of cardiac abnormalities has been investigated in a study on different types of limb–girdle muscular dystrophy based on clinical investigation, echocardiograms, and electrocardiograms. In the patients with LGMD2A, there were no cardiac abnormalities [50]. It was noted that this was not age-related or due to a milder phenotype. However, the absence of cardiac involvement was attributed to the calpain 3 expression deficiency in cardiomyocytes, even if the *CAPN*3 gene transcripts were present (*CAPN3* being the gene with mutation results in LGMD2A) [50]. Cardiac involvements in LGMD patients differ from subtype to subtype. For example, LGMD1B, a type similar to Emery–Dreifuss regarding the mutated gene, is characterized by late dilated cardiomyopathy and arrhythmia [51]. On the other side, LGMD2B, caused by mutation in dysferlin gene, is known for the fluctuating onset of dilated cardiomyopathy [52]. Subtypes LGMD2C-F and LGMD2I—generated due to dysfunction in the dystrophin–glycoprotein complex—are correlated with progressive dilated cardiomyopathy [49]. In the group of patients with LGMD2I, the results indicated a higher prevalence of cardiac abnormalities in males than in females. A correlation between differential expression of sarcoglycan complex proteins and cardiac involvement was underlined [50].

There have also been four sarcoglycan genes reported (*SGCA*-LGMDR3, *SGCB*-LGMDR4, *SGCD*-LGMDR5 and *SGCG*-LGMDR6) that are correlated with autosomal recessive LGMD. One of the main roles of these glycoproteins is to control the integrity of the muscle membrane during contraction and relaxation. These types of LGMD patients exhibit progressive proximal muscle weakness that usually starts before the age of 10. A study on 396 patients from 16 different countries showed that 19% of these patients presented cardiac involvement. The most frequent cases of cardiac involvement were found in LGMDR4. An interesting correlation was made between the apparition of cardiac involvement and the extent of disease for patients with LGMDR4 and LGMDR6 [53].

### 3.7. Myotonic Muscular Dystrophy

Myotonic muscular dystrophies are autosomal, dominant, inherited disorders represented by progressive muscle weakness and skeletal muscles delayed relaxation (myotonia). There are two types of myotonic muscular dystrophies: Type 1 and type 2. Myotonic dystrophy type 1 (DM1), also known as Steinert’s disease, affects both the skeletal and the smooth muscles, as well as multiple systems such as endocrine and central nervous systems; the onset age of the disease varies from infancy to adulthood. Even though the molecular pathway of the disease in not completely understood, it is known that the genetic defect results from an unstable DNA sequence of the *myotonic dystrophy protein kinase* (*DMPK*) gene [2]. There is a phenomenon that takes place associated with children from DM1 parents known as ‘anticipation phenomenon’. This phenomenon takes place in successive generations and is characterized by a higher phenotype severity alongside a decrease in onset age [54]. Based on the severity, three phenotypes have been identified: Mild, classic, and congenital DM1. The first one is defined by mild myotonia and cataract; patients suffering from mild DM1 have an unaffected life span. Classic DM1 is similar to the mild one, with the addition of muscle weakness and cardiac involvement; patients suffering from classic DM1, usually in their adulthood, may be physically disabled and their life span is affected. The latter case is characterized by severe generalized muscle weakness, usually from birth; it is common for the patients to encounter respiratory insufficiency, mental retardation., and early death [5]. Myotonic dystrophy type 2 (DM2) is characterized by slowly progressive proximal muscle weakness, early development cataracts, and an affected endocrine system. The genetic defect results from an unstable DNA sequence of the *cellular* *nucleic acid-binding protein* (*CNBP*) gene. Between DM1 and DM2, DM2 is the milder version of myotonic muscular dystrophy, with later disease onset. Cardiac abnormalities are also involved, such as arrhythmias, dilated cardiomyopathy, left ventricular dysfunction, or conduction disorders [55,56].

For both types of myotonic muscular dystrophy, it has been suggested that cardiac involvement is associated with gap junction and the overexpression of the calcium or sodium channel protein [57]. Although similar, it has been observed that cardiac involvement is more severe in the case of DM1 and has an earlier onset [49].

When discussing cardiac involvement in young patients with DM1, it can be determined as soon as the patient is 20 years of age. Over 60% of adult patients experience conduction disorders. Echocardiography and cardiovascular resonance imaging can be useful techniques in order to investigate ventricular dysfunction and structural changes. It is not a common feature for DM1 patients to experience sudden death; however, cases have been reported. It is believed that one of the reasons for that may be ventricular tachycardia [58]. Permanent pacemaker implantation has been established as being beneficial even for asymptomatic patients with abnormal electrocardiograms [42]. Moreover, myocardial fibrosis could be highlighted by late gadolinium enhancement cardiovascular magnetic resonance [59]. A study on early cardiac diagnosis in DM1 patients revealed that multiparametric cardiac magnetic resonance can detect interstitial fibrosis in asymptomatic patients, therefore being of high importance in discovering myocardial abnormalities [60].

### 3.8. Oculopharyngeal Muscular Dystrophy

Oculopharyngeal muscular dystrophy is a genetic disorder characterized by an adult-onset and proximal muscular weakness, mainly of the eyelids (ptosis) and throat, the latter leading to dysphagia. Even though oculopharyngeal muscular dystrophy has a progressive nature, there are rare cases when the patients are actually wheelchair-bound. The disease is determined by a mutated gene, *poly(adenylate)-binding protein nuclear 1* (*PABN1*), which encodes a protein found all through the body. Although it is a slowly progressive disorder, the progressive rate varies from patient to patient. The main cause of death in patients who have oculopharyngeal muscular dystrophy is aspiration pneumonia combined with starvation. The disorder is not gender-dependent and cardiac abnormalities are not characteristic [61,62]. 

## 4. Conclusions

Muscular dystrophies are a big group of disorders that progressively affect the skeletal muscles. However, the main difference between all these types of muscular dystrophy comes from the age of onset, the targeted muscles, progression rate, type of gene mutated, and several other parameters that make them distinguishable. The majority of muscular dystrophies also exhibit cardiac abnormalities; in some cases, these are critical and determine the disorder’s onset. Cardiac involvement can be present with symptoms ranging from mild to highly severe; therefore, cardiac imaging is one major step that needs to be taken when monitoring muscular dystrophies.

## Figures and Tables

**Table 1:** Recent (last 3 years) research on cardiac involvement using cardiac imagistic techniques.

Type of MD	Cardiac Involvement	Imagistic Technique	No. Patients (Age of Patients)	Reference
Becker and Duchenne MD	- LVEF - Heart wall motion abnormalities - Increased LV end-systolic/diastolic dimensions	Echocardiography	130 (39 ± 15.7 years)	[19]
	- Assessment of LV function - Progression of myocardial fibrosis	Cardiac magnetic resonance imaging	76 (12.1 ± 2.7 years)	[63]
	- Myocardial fibrosis	Late-gadolinium enhanced cardiac magnetic imaging	35 DMD/33 BMD(17.4 ± 6/18.3 ± 3.9 years)	[64]
	- Progression of LV dysfunction and myocardial fibrosis - LVEF	Cardiac magnetic resonance imaging	34 (IQR, 6.4–15.9 years)	[65]
	- Heart rhythm disturbances - Mild LV dysfunction - Mildly impaired LVEF - Cardiomyopathy	Electrocardiography Echocardiography Cardiac magnetic resonance imaging	34 (10.5 ± 1.5 years)	[66]
	- Myocardial dysfunction	Myocardial strain imaging	25 (14.8 ± 3.1 years)	[20]
	- Heart rhythm abnormalities	Holter monitoring	N/A	[24]
Congenital MD	- First degree atrioventricular block - Atrial tachycardia and arrhythmias - Premature ventricular contractions - LVEF - Early ventricular fibrosis	Electrocardiography Holter monitor Cardiac magnetic resonance imaging	3 case reports	[33]
	- Enlarged right atria with dilated coronary sinus - Right or/and LV hypertrophy - Hyper-trabeculated LV - LVNC	Fetal echocardiography Electrocardiography Echocardiography	1 (4 months)	[34]
Distal MD	- Takotsubo syndrome - Reduced systolic function - Regional heart wall motion abnormalities - Low LVEF - Atrial fibrillation - Valvular heart disease - Dilated cardiomyopathy	Electrocardiography Echocardiography Cardiac ventriculography Cardiac magnetic resonance imaging	1 case report	[67]
	- LV hypertrophy - Case of distal MD without cardiac involvement	Echocardiography Holter monitor Magnetic resonance imaging	18	[68]
	- Dilated cardiomyopathy - LV dysfunction - Cardiac arrhythmia - Atrial fibrillation - Bradycardia	Cardiac ultrasound Electrocardiography	244 (5–74 years)	[69]
Emery–Dreifuss MD	- Atrial fibrillation - Bradycardia - Severe dilatation of LV - Cardio-embolic stroke	Electrocardiography Echocardiography Cardiac magnetic resonance imaging	1 (23 years)	[70]
	- Sinus rhythm with alterations of ventricular repolarization - Asymmetric hypertrophic cardiomyopathy	Electrocardiography Echocardiography	1 (18 years)	[71]
	- Atrial flutter - Biatrial enlargement and atrial standstill - Dilated cardiomyopathy	Electrocardiography Echocardiography	1 case report	[72]
	- Atrial fibrillation - Sinus rhythm - Increased LVEF - Increased cardiothoracic ratio	Electrocardiography Echocardiography Chest X-ray	1 (36 years)	[73]
Facioscapulohumeral MD	- Incomplete right bundle branch block - Heart failure - Atrial fibrillation - No cardiomyopathy	Electrocardiography Holter monitor Echocardiography	56 (21–86 years)	[47]
	- Focal and diffuse myocardial injury - Myocardial fat infiltration - LVEF	Electrocardiography Holter monitor Cardiac magnetic resonance imaging	52 (48 ± 15 years)	[74]
Limb–Girdle MD	- LV hypertrophy - Dilated LV - Reduced LVEF - Dilated cardiomyopathy	Electrocardiography Echocardiography Holter monitor Cardiac magnetic resonance imaging	2	[75]
	- Secondary cardiomyopathy - Ventricular arrhythmias - Heart failure - Premature ventricular complexes - First-degree atrioventricular block - Dilated LV - Reduced LVEF - Mitral regurgitation	Electrocardiography Transthoracic echocardiography Cardiac magnetic resonance imaging	1 (39 years)	[76]
	- Single coronary artery	Computer tomography	181 (10.1 ± 4.6 years)	[25]
	- Dilated cardiomyopathy - Atrial fibrillation - Left bundle branch block - Low LVEF	Transthoracic echocardiography	N/A	[26]
Myotonic MD	- Low LVEF - Patchy myocardial fibrosis - Myocardial inflammation - Myocardial dysfunction	Cardiac magnetic resonance imaging Electrocardiography Holter monitor	N/A	[29]
	- Asymptomatic patients - Myocardial inflammation - Myocardial fibrosis	Cardiac magnetic resonance imaging Transthoracic echocardiography	115(1–68 years)	[31]
	- LV dysfunction - Low LVEF - Atrial fibrillation	Cardiac magnetic resonance imaging Electrocardiography	N/A	[42]
	- Mid-wall myocardial fibrosis - Atrioventricular block	Late-gadolinium enhanced cardiac magnetic imaging Electrocardiography	52 (41 ± 14 years)	[59]
Oculopharyngeal MD	- Right/left bundle branch block - Heart failure - Non-sustained ventricular tachycardia	Transthoracic echocardiography Electrocardiography	1 (35 years)	[77]

MD: Muscular dystrophy, LV: Left ventricular, LVEF: Left ventricular ejection fraction, LVNC: Left ventricular non-compaction cardiomyopathy, IQR: Interquartile range.

## Data Availability

Not applicable.
